# The Case of the Discharged Consumptive

**Published:** 1918-11-09

**Authors:** 


### THE PERMANENCE OF PENSIONS.

### The Case of the Discharged Consumptive.

We have always kept a watchful eye upon the conditions attached to pensions, and have been careful to publish one by one, as each appeared, any insidious devices for abating or rescinding them. Our readers will recall these in their order. The first was a colonel's suggestion that the disabled, when trained to perform some productive work, might "feel too proud" to continue to accept the reward for their past services. To this we replied that "the disabled should stick to their pensions as fast as they formerly stuck to their guns," and the disgraceful proposal was shamed into silence. There have been several since. Sir Frederick Milner could supply a complete list, for he has been patriotically alert to scotch each of the hydra-headed brood as it appeared. Among the later devices is that which refuses permanent pensions to officers and men discharged in consequence of tuberculosis, the one disablement of all others for which permanent provision must be made. This cruel limitation to a temporary pension only has led to the formation of the Disabled Officers' Society. This has already petitioned Mr. Hodge, the Pensions Minister, on behalf of consumptives discharged from all the Services, whether, we understand, officers or men or naval ratings. The petition claimed a permanent pension, not temporary retired pay, and pointed out that neither officers nor men could maintain themselves and their families on, as a member of the Society put it lately in the *Weekly Dispatch*, "the meagre allowances" granted by the Ministry of Pensions.

The next point emphasised by the petitioners was the unfairness of the Ministry's practice in re-assessing the degree of disablement of the tuberculous soldier on his dicharge from a sanatorium. A sanatorium exists to prevent the spread of the disease, if and when early cases are brought under its treatment; to arrest its development in later stages, and in theory (for the institutions for this purpose hardly can be said to exist even to-day) to provide separate accommodation for the highly infectious, the advanced, and the dying. The sanatorium does not profess to cure. That word has not been publicly proclaimed in a general sense, except on the political platform, when the sponsor of the insolvent Insurance Act temporarily lost his head. Experienced tuberculosis officers do not talk of "cure"; that is reserved for quacks, the periodic enthusiast, the demagogue, and now, it would almost seem, the unfortunate Mr. Hodge. His Department apparently is seeking in the word "cure" a loophole by which it may evade its full responsibility for giving permanent pensions to ex-soldiers who have contracted a chronic and permanent disease! The petitioners considerately pointed out that, after discharge from a sanatorium, a careful life for two years was medically essential if relapse was to be prevented. The assessors appointed by the Ministry to "re-assess" (that is the official phrase) the degree of disablement upon the man's discharge from a sanatorium would do well to remember that their eyes should be fixed rather on the danger of "relapse" than on the degree of arrestment. They may be sure that the criticism of the public will rise to fury if many *relapsed cases occur among men who have been previously deprived of their pensions*. Before that fury the most bureaucratic of Ministers will have to bow, and questions in Parliament will soon elicit the names of the assessors who were in such haste to take a conveniently optimistic view, and the names, too, of those responsible for not checking the "judgments" of the assessors. The public will remind these gentlemen in the newspapers, on the platform, and persistently on the floor of the House of Commons, that the discharged tuberculous soldier is always a marked man when seeking employment, that a degree of ostracism is always threatening him, and that the allowance which he may have does little more than keep him in a constant state of anxiety: in the first place because of its amount, and in the second because of its insecurity. Finally, the unfortunate man cannot even emigrate (however convenient that would be to the Ministry of Pensions), because the ports of the otherwise friendly Colonies are, generally speaking, closed to *him*.

Confronted by such enormous arguments as these, Mr. Hodge was, briefly, adamant. We say Mr. Hodge because, even if it is the Treasury rather than himself, it is his business to persuade the Treasury, just as it is the business of the public to convince them both, perhaps (since both the Pensions Ministry and the Treasury are controlled by politicians), by a run of independent candidates at the polls! It is true that Mr. Hodge said that when the disease is deemed to reach "its final condition" a permanent pension might be granted, but he forgot that men do not need a permanent, or other, pension in the grave. It is true that he said that, if the consumptive officers were more generously treated, similar treatment would have to be accorded to the men, a position which, with the spectre of the Treasury behind his chair, seemed to him to reduce the argument for fairness to absurdity! These jokes and these absurdities, however, are too grim, and their grimness in the long run will decide the matter. Meantime, the Disabled Officers' Society refused to split the Service by demanding preferential treatment for its members. It insisted on fair treatment for all men discharged through contracting a disease which is indifferent to the rank or ribbons of its victims.

The case of the petitioners was so tremendous that the Pensions Minister had to fall back on the official skeleton which is produced only when a Government Department is in desperate straits indeed. He pointed out, with becoming awe, that the arguments of the petitioners were without the scope of the terms of the Royal Warrant. Just

**The Permanence of Pensions**—(*continued*).

think of that! The Royal Warrant had not "envisaged" (this seems to be the only verb august enough to describe the workings of the Royal Warrant's mind), had not envisaged the situation described by the petitioners. The bare hint, the merest implication that the Royal Warrant could be altered, might *have* to be altered very probably, the faintest of these suggestions was unthinkable. When, in a further communication, the substance of them was urged by letter upon Mr. Hodge, that letter was not considered to merit a reply. When a request was made, after a suitable delay, for some acknowledgment, a secretary regretted that nothing further could be done. We shall see. The mention of the Royal Warrant is the official equivalent of "hands up," and of the abandonment of the official case as hopeless. This point towards a different policy is a step familiar to all, from Florence Nightingale downwards, who have brought recalcitrant Government Departments to their knees. The Disabled Officers' Society should feel much encouraged. It has caused the enemy to cry mercy. The *coup de grâce* will be given in time by the Society, through the House of Commons, and through public support elsewhere.

